# Valorizing the 'Irulas' traditional knowledge of medicinal plants in the Kodiakkarai Reserve Forest, India

**DOI:** 10.1186/1746-4269-5-10

**Published:** 2009-04-14

**Authors:** Subramanyam Ragupathy, Steven G Newmaster

**Affiliations:** 1 Biodiversity Institute of Ontario Herbarium, University of Guelph, Guelph, Ontario, N1G 2W1, Canada

## Abstract

A mounting body of critical research is raising the credibility of Traditional Knowledge (TK) in scientific studies. These studies have gained credibility because their claims are supported by methods that are repeatable and provide data for quantitative analyses that can be used to assess confidence in the results. The theoretical importance of our study is to test consensus (reliability/replicable) of TK within one ancient culture; the Irulas of the Kodiakkarai Reserve Forest (KRF), India. We calculated relative frequency (RF) and consensus factor (F_ic_) of TK from 120 Irulas informants knowledgeable of medicinal plants. Our research indicates a high consensus of the Irulas TK concerning medicinal plants. The Irulas revealed a diversity of plants that have medicinal and nutritional utility in their culture and specific ethnotaxa used to treat a variety of illnesses and promote general good health in their communities. Throughout history aboriginal people have been the custodians of bio-diversity and have sustained healthy life-styles in an environmentally sustainable manner. However this knowledge has not been transferred to modern society. We suggest this may be due to the asymmetry between scientific and TK, which demands a new approach that considers the assemblage of TK and scientific knowledge. A greater understanding of TK is beginning to emerge based on our research with both the Irulas and Malasars; they believe that a healthy lifestyle is founded on a healthy environment. These aboriginal groups chose to share this knowledge with society-at-large in order to promote a global lifestyle of health and environmental sustainability.

## Background

A mounting body of critical research is raising the credibility of Traditional Knowledge (TK) in scientific studies and natural resource management. The lack of recognition of the place and value TK in science has prevented real engagement of this knowledge in scientific endeavours including nutrition, medicine, environmental assessment and resource management practices [1]. One explanation is the lack of validation using quantitative analyses that give some measure of confidence and methods for replication. Clearly there is some asymmetry between scientific and TK, which requires new approaches to the relationship between scientific and TK. We must consider some criteria for negotiating and legitimating the validity of knowledge. Science is based on the evolution of knowledge, which is testable and ultimately generalisable, mobile and globally meaningful [2].

To gain credibility, scientific studies that utilize TK must be reliable (refutable) and designed so that they can be replicated. The need for full disclosure in science demands quantitative measures of the reliability to be established. In ethnobotanical studies, this was established by Trotter and Logan [3] who developed a quantitative method to evaluate consensus among informants in order to identifying potentially effective medicinal plants. Consensus analysis provides a measure of reliability for any given claim providing refutable evidence. This evolved into the "Factor of informant Consensus" (FIC) in order to quantitatively evaluate the degree of selection of certain plants for a particular utility (eg, healing an ailment). One of the traditional intentions of FIC is to test the homogeneity among informants' knowledge [3-6]. Researchers use consensus analysis to test falsifiable hypotheses concerning informant selection and use of plants [5,7], as a decision making factor [4,8,9], to weight the relative importance of TK [10], and to estimate the competence of informants [11-13]. These studies have gained credibility because their claims are supported by methods that are repeatable and provide data for quantitative analyses that can be used to assess confidence in the results.

India is rich in ethnic diversity and traditional knowledge (TK) that has resulted in a considerable body of ethnobotanical research, of which one study has revealed a deep understanding of medicinal plants supported by high consensus. There are over 537 different aboriginal groups in India with extensive knowledge of plants [14-16]. Many qualitative surveys have recorded detailed utility of specific plants for many aboriginal groups such as the Malasars [17], Malamalasars [18], Malayalis [19-22], Irulas [23-27], Gonds [28], Koysd, Konda reddis, Valmikis, Koyas, Chenchus, Lambadis, Jatapus, Savaras, Bagatas, Kammaras, Khondas, Nukadoras, Porjas, Jatapus [29], Paliyar [30], Kanikar [31], Todas, Kotas [32,33], Kattunayakas [34], Apatani [35] and Chellipale [36]. Recent research by Ragupathy et al. [37] presented the first quantitative research in India that considers variation in TK among informants using a quantitative consensus analysis. Their research discovered high consensus in TK and new insights in medicinal utility by the Malasars towards understanding their vital well being and good health [37]. This research provided several alternative hypotheses for consensus of TK that may be low when considering different spatial scales [38], unreliable TK, informant bias, localized remedies, variability in local ethnotaxa, availability of multiple traditional remedies [39-41] or recent replacement by pharmaceutical supplements [42].

The theoretical importance of our study is to test consensus (reliability/replicable) of TK within one ancient culture; the Irulas of the Kodiakkarai Reserve Forest (KRF) in the Coromandal coast of Thanjavur district, India. We chose to work with the Irulas of India, because they are known to be exceptional healers and keepers of TK of the flora in the coastal forest [24] and there is limited research on the Irula's TK [43]. Furthermore, the Irulas are an example of a culture that has preserved a highly diverse area ecosystem that sustains their healthy life-styles [23,27]. We hypothesize that consensus of TK of specific plants used for different illness categories are high indicating reliable TK among informants at a local scale (within one localized aboriginal group or community – Irulas of the KRF), because it has been used within their culture without interruption for many generations. This hypothesis concurs with the results of our study of the Malasars TK of medicinal plants. Scientific inquiry demands that research can be replicated in order to substantiate claims of medicinal utility within any aboriginal culture. Alternatively, consensus of TK may be low at local scales [37,39] because of i) unreliable TAK, ii) informant bias, iii) local remedies; certain villages may have unique uses for plants, iv) variability in local ethnotaxa; certain communities may have found variants or ecotypes for some plants that result in unique qualities that are of particular use at only a local scale, iv) use of pharmaceutical supplements; the availability of modern pharmaceuticals for a particular ailment may result sporadic use of traditional remedies, and v) availability of multiple remedies; there may be groups of plants and therefore several remedies available that are preferentially selected by individual healers for various utilities (eg, healing some ailment), thus indicating the potential biological activity for a group of plants [39]. These groups may represent Linnaean taxa (ie, genus or family) that share similar biological processes, or aboriginal classifications may group plants that serve a similar utility [40,41].

### The Irulas and their land – Ethnography

The Irulas are as small tribal community that is part of the Dravidian language group that is spoken in south-eastern India. They are recognized as a Scheduled Tribe (ST) by the Government of India [44]. Most ethnic populations of southern India are linguistically Dravidian [45,46]. The Irulas belong to the Negrito (or Negroid) race, which is one of the six main ethnic groups that add to the racial mosaic of India [47]. Negroids from Africa were the oldest people to have migrated to India [47]. The structure of their tribal dialects is slightly different from modern Dravidians [48]. The Dravidians of South India speak Tamil, Telugu and Malyalam, but the Irulas in KRF speak a different dialect of Tamil, which is distantly related to other Dravidian Tamil. The Irulas, the largest Tamil speaking adivasi (Aboriginal) tribe in Tamil Nadu, found themselves virtually bonded labourers in 1976 when the Forest Protection Bill deprived many of their traditional lifestyle [49]. The Irulas started moving to the neighbouring villages in hope of rebuilding their lives [50]. The origin of the word "Irulas" is not clear. Some surmise that it is derived from the Tamil word, 'Irul', implying the dark complexion of the Irulas often being spotted by villagers as distant silhouettes in the forests, and supporting their local name, "the Forest People" [51].

The culture of the Irulas has changed little over the last thousand years. Their staple food consists of minor millets, grain legumes, and wild yams supplemented with rice. They do not practice agriculture and therefore wholly depend on forest produce and wild animals like wild bore, rabbit, rat, deer, wildcat and fish. The Irulas settlements are located within or on the edge of the forests and consist of tiny scattered huts. The community is divided into several exogamous clans. Marriage between inhabitants of different hamlets is generally encouraged and patrilocal residence is commonplace. More recently, some Irulas go to local villages to trade or sell honey, honey wax, firewood, wild fruits, yams, wild Carissa, Manilkara and Solanum berries and other native herbal products. This supports a productive and localized non-timber forest product (NTFP) industry, which supports many families. The Irulas trade NTFPs for local farm produce such as rice, potato, eggplant, tomato, tea, cookies and spices. Other occupations of the Irulas include intermittent farm labour and the legendary profession of snake charming. They have distinct cultural and religious practices, including worship of the Goddess Kanniamma, and appointment of Pujari 'priests' to serve their communities.

### Biogeography of the Study Site

**Figure 1:**
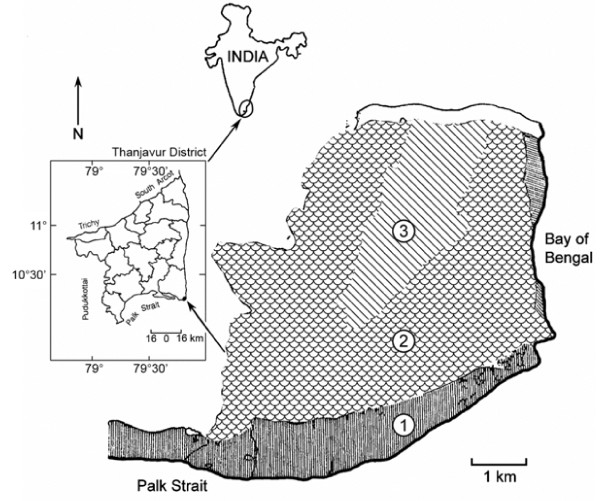
**Kodiakkarai Reserve Forest (KRF) located on the Coromandel Coast of Thanjavur district, Tamil Nadu, East Coast of India (Note: 1 = wetland; 2 = dry evergreen forest; 3 = grassland)**.

Kodiakkarai Reserve Forest (KRF) is a dry evergreen tropical forest located on the Coromandel Coast of Thanjavur district, Tamil Nadu, South India (Figure 1), 325 km south of Madras city, the capital of Tamil Nadu. The reserve covers an area of about 25 sq. km [24]. Geographically the KRF lies at latitude 10° 18' N and longitude 79° 51' E, and is bordered by the Bay of Bengal to the east and Palk Strait to the south. The elevation ranges 25 to 50 m above mean sea level. Like most other costal areas in Thanjavur District, the climate of KRF is warm and moist. Monthly mean temperature of the area is between 24°C and 32°C, with maximum temperatures rising to 41°C and minimum temperatures near 29°C. Monthly mean percentage of humidity is between 60% and 80%, with a maximum of 92.3% during October and a minimum of 69.6% in April. The annual precipitation ranges from 1000 – 1500 mm with the wet South West monsoon occurring from June until September [52]. The KRF is often impacted by serious erosion during the Southwest monsoon because of the heavy rainfall in the Western Ghats washes loose soil from the hills that invariably feeds the Cauvery River and empties into the Indian Ocean [24].

The diversity of wildlife in the KRF is rich and includes many tropical species of monkeys, jackals and foxes. The coastal belt of dry evergreen forest at Kodiakkarai hosts large populations of spotted deer, antelopes and wild pigs. Crocodiles once existed in large numbers but they have now dwindled due to over-hunting. The swamps of Point Calimere within the KRF contain over 240 bird species, including large populations of flamingos and a rich diversity of stints, ducks, herons, painted storks, teals, terns, pelicans, egrets, sand pipers and Mongolian plovers [53]. The plant diversity includes more than 370 species of vascular plants within three main floristic regions: dry evergreen forest (Figure 1), wet forests (mangroves) and grasslands [24].

## Methods

### Ethnobotanical survey and consensus analysis

Our survey protocols are identical to our previous study [37] of the Malasars in the Velliangiri Hills of India. These methods follow standard methods for interviews, data confirmation and field observation as suggested by Bernard [54]; Etkin [55]; Pelto and Pelto [56]; Alexiades [57]. To elucidate community domains and determine differences in TK among the informants we cross checked with new informants to determine the percent frequency of a particular response within our survey. The local 'headman' helped to us record general information on the local customs, habits and beliefs, information on the surrounding area and individuals who are knowledgeable of the local flora and could serve as informants [58].

The consensus analysis utilized the informant's TK as defined by the surveys. Local informants (ie., traditional healers) having practical knowledge of plant medicinal utility of the KRF were interviewed during April 2005 – January 2007. During the course of the study, about 7 field trips were conducted in the study area. Surveys were conducted by a stratified random selection of informants, based on methods suggested by Schultes [59,60], Jain [15] and Bernard [54]. Successive free listing was used to interview 100 informants providing data for the consensus analysis. The informants were selected following standard interview protocols [54,61,62]. We interviewed over 120 informants from which we selected 80 informants that were equally distributed among four different age categories; elders, middle aged, teenagers, < 10 years. During these interviews we documented all possible information about a specific plant and then sorted this information according to utilitarian perspective, local name or ecology. The informants were given limited time and there was no differentiation among gender. We also asked the informants to group the plant specimens into different illness categories that were predefined from previous research [37], and through consultation with the headman and individual informants. We requested all informants to collect specimens of the common plants they knew or to show the plant species on site. The questionnaires were used to obtain information on medicinal plants with their local names, parts used, mode of preparation and administration.

The relative frequency (RF) of TK from each individual ethnotaxa from the interviews was calculated to determine a quantitative value for validating TK of ethnotaxa utility (eg, remedy of choice). Although being named by three informants is the most common cut-off point to establish consensus [63,64], we choose to calculate the RF for all TK with respect to each ethnotaxa.

Calculation of a consensus factor (F_ic_) for testing homogeneity of informant's knowledge followed methods of Trotter and Logan [3]. A consensus factor of F_ic _is given by:



The factor provides a range of 0 to 1, where a high value acts as a good indicator for a high rate of informant consensus. Nur is the number of use-reports of informants for particular illness usage, where a use-report is a single record for use of a plant mentioned by an individual, and Nt refers to the number of species used for a particular illness category for all informants. The majority of illness types are grouped into predefined ethno/economic botany categories [4,65], with the additions of a few other illness categories (Table 1) that were commonly mentioned during our interviews because they were prevalent in these communities. The use of "general categories" is adopted here as recommended by other ethnobotanical researchers [4,65]. These 30 illnesses were sorted into 12 usage categories (Table 1). All of the illness types were translated as best as possible from the Irulas description of the illness/symptoms to known biomedical terms, with few exceptions (eg, spiritualism – keep family wellness/repel evil).

**Table 1:** Irulas ailments grouped by illness category

Illness Category	Biomedical/English Term	Irulas Term
Dermatological	Skin disease	*Thol viyathi*
	Skin appearance	*Palapalappu*
Diabetes	Diabetes	*Chakari noie*
Fever	Fever	*Kaichal*
Gastrointestinal	Diarrhoea	*Vaitru po'kku*
	Purgative	*Vaitru po'kku*
	Intestinal worms	*Vaitru poochi*
General health	Complementary food	*Siru ounavu*
	Antidotes	*Visha murichi*
	Anti mosquito	*Nochi vadai*
	General medicine	*Podhu maruthuvam*
	Cold	*Shzali*
	Antibiotic	*Ethir nachu*
	Lethargy	*Kayakalpa*
	Appetite	*Pasi edupu*
	Hypothermia	*Soodu thanippu*
	Blood Flow	*Ratha o'ttam*
Hydrocele	Hydrocele	*Virai veengi*
Infection	Wounds	*Kayam*
Jaundice	Jaundice	*Manjal kamalai*
Leprosy	Leprosy	*Thozhu noie*
Pain	Headache	*Thalai vali*
	Eye pain	*Kan vali*
	Earache	*Kadhu vali*
	Toothache	*Pal vali*
	Pregnancy pain	*Prasava vali*
	Body ache	*Udambu vali*
	Rheumatism	*Moottu vadham*
Respiratory	Asthma	*Izhuppu*
	Cough	*Irummal*
Spiritual	Wellness	

### Botanical documentation and preservation

All plants utilized in the interviews were collected as vouchers for confirmation of identity using the Irulas classification system [66]. The Linnaean identities were designated by comparing the specimens with the authentic type specimens in herbaria, and by referring to recent taxonomic monographs and revisions. The botanical nomenclature followed that of Flora of Tamil Nadu, India Series I Analysis [67,68]. Live species were propagated and can be found in the E.K. Janakiammal Etnobotanical Garden, Botany Field Research Laboratory, University of Madras, Maduravoyal, Chennai. Voucher specimens are deposited in the herbarium of the University of Madras, Chennai, and the herbarium of Botanical Survey of India, Southern Circle (MH).

## Results and discussion

### Consensus of the Irulas TK of medicinal plants

Our research indicates a high level of consensus of TK of medicinal plants within the Irulas community. In our study, the relative frequency (RF) of TK from each individual ethnotaxa from the interviews was high (mean RF = 0.92 ± 0.04). The informant consensus of medicinal plant usage resulted in F_ic _ranging from 0.70 to 1.00 per illness category (Table 2). The average F_ic _value for all illness categories was 0.85, indicating a high level of informant consensus compared to similar studies from other countries [4,38]. In the literature, high informant consensus was also recorded among studies with many illness categories such as a study with the Malasars (F_ic _= 0.71) in India [37] and several studies focused on specific illness categories: the snakebite healers of Kamba in Africa (F_ic _= 0.88) [69]; and treating 'mich' or febrile diseases (F_ic _= 0.80) [70], and respiratory disorders (F_ic _= 0.88) among Inuit in Nunavut [54]. We recently published [37] the first study to utilize a consensus analysis in ethnobotanical research within India. This research investigated the Malasars occupying the forests of the Velliangiri holy hills in the Western Ghats, South India. The results of this research revealed 95 ethnotaxa that are utilized by the Malasars for medicinal and general health purposes of which most had high consensus (F_ic _> 0.80) for some ailments such as respiratory and jaundice. Our current research is exploring the consensus of TK with respect to the same ethnotaxa across spatial (beyond local geographic villages) and cultural scales (among different cultures such as the Irulas and Malasars).

**Table 2:** Ethnobotanical consensus index for traditional medicinal plant use categories

Illness category	Number of use-reports (N_ur_)	Number of Taxa (N_t_)	Informants' consensus index factor (F_ic_)^a^
Dermatological	12	3	0.82
Diabetes	7	1	1.00
Fever	12	4	0.73
Gastrointestinal	17	5	0.75
General health	61	19	0.70
Hydorcoele	6	1	1.00
Infection	13	4	0.75
Jaundice	6	1	1.00
Leprosy	5	1	1.00
Pain	46	14	0.71
Respiratory	8	2	0.86
Spiritual	13	2	0.92

^a^F_ic _= N_ur_-N_t_/(N_ur_-1), providing a value between 0 and 1, where high value indicates a high rate of informant consensus.

### Diversity of ethnotaxa utilized by the Irulas for medicine

**Figure 2:**
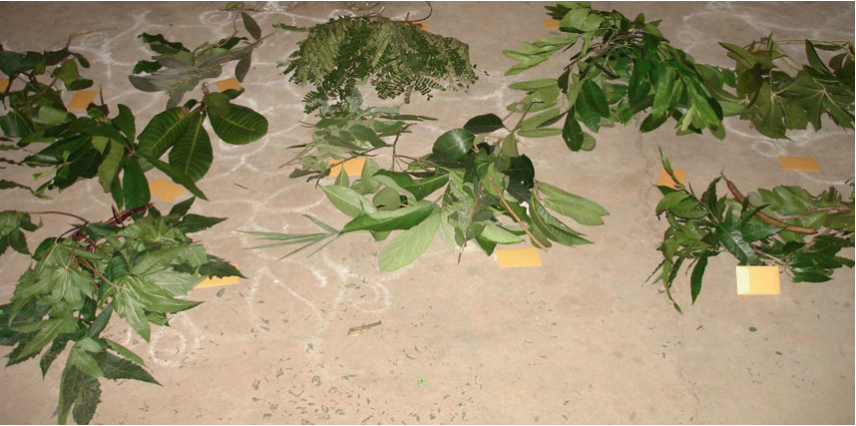
**Sorting of ethnotaxa by the informants**.

**Figure 3:**
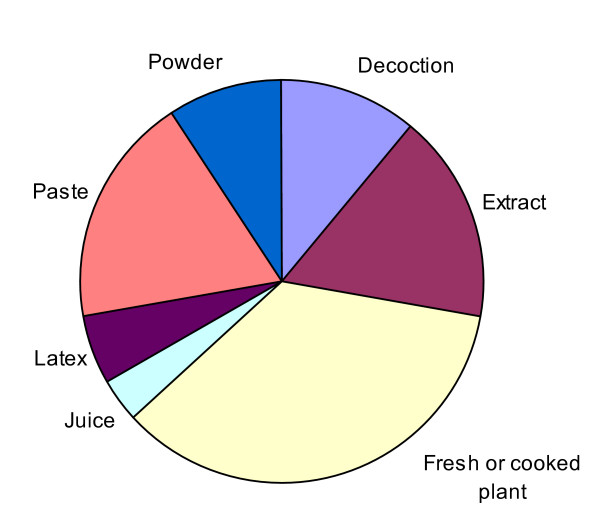
**Categories of Irulas mode of utilization for various ailments**.

The Irulas revealed a diversity of plants that have medicinal and nutritional utility in their culture. During this study the Irulas informed us of 53 ethnotaxa that have considerable utility in medicinal and general health care purposes within their culture (Table 3; Figure 2). This body of TK concerning medicinal and nutritional plants was supported by high consensus (mean F_ic _= 0.85 ± 0.04). We provide the medicinal utility and method of preparation for each ethnotaxa in Table 3. The 53 common ethnotaxa were classified within Linnaean taxonomy to represent 52 species distributed in 51 genera belonging to 33 families of plants. The Irulas prefer to utilize species from primary or secondary dry evergreen coastal forests of Coromandal Coast of Thanjavur district, Southeast India, rather than the weedy species from disturbed areas (Figure 1). The most common plant families in the study were Euphorbiaceae (4 species) and Fabaceae (9 species) (complete list of families is given in Table 3). Herbs (10 species) were the most common functional group of plants, followed by shrubs (13 species), climbers (10 species) and trees (17 species). A variety of plant morphological structures were utilized as medicine by the Irulas of which leaves were used most frequently, followed by roots, bark, seeds, whole plants, flowers, fruits and latex/sap. The preparation for utilization of these plant parts can be grouped into several categories based on the mode of preparation; decoction, extract, fresh or cooked plant, juice, latex, paste, and powder (Figure 3).

**Table 3:** Irulas medicinal utility of the flora in the Kodiakarai Reserve Forest (KRF)

Botanical Name and Family	Tamil Lexicon	Method of Preparation and Medicinal Uses
Acanthaceae		
	*Auduthoda*	Extracts of root, bark, leaves, and flowers used for cough. Dried leaves emhaled for rapid recovery from asthma. Fresh flowers kept over the eyes to relieve eye pain.
Amaranthaceae		
*Achyranthes aspera *L.	*Nagarasi*	Extract of leaves along with palm jaggery fed to new born babies. Inflorescence scratched against mother breasts to increase lactation.
*Aerva lanata *(L.) Juss.ex Schult *Celosia argentea *L.	*Kannupila & Pannaipoo*	Whole plant harvested from the forest or sacred groves during festival day (Farmers Thanksgiving Day), which is used spiritually for healing sour necks of bull.
*Aristolochiaceae*		
*Aristolochia indica *L.	*Perumanthikodi*	Root tubers ground with a little water, mixed with rice or cow's milk to cure a fever.
Arecaceae		
*Borassus flabellifer *L. *Phoenix sylvestris *(L.) Roxb.	*Panai* *Injai*	Toddy tapped from the inflorescence. Brushes and ropes prepared from leaf sheaths. Leaves used for thatching. Meristematic portion of the stem eaten by pregnant women from 9th month onwards for relief from abdominal pain.
Asclepiadaceae		
*Gymnema sylvestre *R.Br.	*Sirukurinjan & hakarikolli*	Tender fresh leaves and dried powder are used to cure diabetes. Cooked leaves may also be used in meals to treat diabetes.
Asteraceae		
*Tridax procumbens *L.	*Vettukayapoondu*	Leaf juice applied on wounds to stop bleeding.
Caparidaceae		
*Capparis sepiaria *L. *Capparis zeylanica *L.	*Thoratti* *Adandai*	Root pulp applied on small wounds and scratches. Leaves mixed with pepper, tamarind and garlic into a paste, which is consumed to increase appetite.
*Cucurbitaceae*		
*Trichosanthes cucumerina *L.	*Peyppadal*	Ripe fruit contains a mucilaginous exudate, which is applied directly on forehead to treat headaches.
*Caesalpiniaceae*		
*Cassia auriculata L*.	*Avarai*	The leaves, flowers, fruits, stems and roots are used to maintain hygiene of the skin and scalp. Every year, the first day of the month of crop harvest is celebrated as the thanks-giving day for the "bull".
Ebenaceae		
*Diospyros ferrea *(Wild.) Bahk. Var. *buxifolia*	*Veeraii*	Fruit improves blood circulation.
Euphorbiaceae		
Acalypha indica L.	*Kuppaimeni*	Decoction of leaves used for ear pain, snake bite and scabies.
Excoecaria agallocha L.	*Thillai*	Latex applied on wounds for antiseptic.
Jatropha tanjorensis Ellis & Saroja	*Katamanukku*	Latex heals wounds.
Phyllanthus amarus Schum. & Thonn.	*Kizhanelli*	Raw branchlets and leaves eaten for 7 days for curing jaundice.
Fabaceae		
*Acacia nilotica *(L.) Willd. ex. Del.	*Karuvelam*	Paste of tender leaves used to control dysentery. Gum powder mixed with the white of an egg, and applied on burns or scalds.
*Albizia lebbeck *(L.) Benth.	*Vagai*	Oil extracted from the seeds smeared on wounds for healing or on the lesions of lepers.
*Caesalpinia bonduc *(L.) Roxb.	*Kazhchikai*	Seeds are made into a paste to treat a hydrocele
*Canavalia lineata *(Thunb.) DC.	*Kozhiavarai*	Roasted seeds of *Mucuna *and *Canavalia *are mixed in equal proportion are boiled and made into a paste, which is soaked in a large container of water. The water is changed periodically for 15 days to remove the poisons. The water is changed until it removes the last trace of yellow colour. The mixture is dried and the white powder is eaten directly with flour or suguar or chilly powder. It cures several disorders and ensures good health.
*Clitoria ternatea *L.	*Sangu pushpam*	Paste of flowers applied to cure infection of eyes and for headache. Entire plant used as antidote for snake bites. Flowers offered to Gods.
*Mucuna pruriens *(L.) DC.	*Poonikali*	See '*Kozhiavarai *' *Canavalia *lineata (Thunb.) DC.
*Pongamia pinnata *(L.) Pierre	*Pongan*	Seeds ground into paste and applied externally for knee and hip joints for rheumatic disease.
*Tephrosia purpurea *(L.) Pers.	*Averi*	Decoction of roots with ginger consumed to relieve headache.
*Thespesia populnea *(L.) Sol. ex Correa	*Poovarasu*	Yellow extract of fruit used for decorating houses.
Gentianaceae		
*Enicostema axillare *(Lam.) Raynal	*Vellarugu*	Secondary roots used as tooth brush to cure toothaches.
Lamaceae		
*Geniosporum tenuiflorum *(L.) Merr. *Leucas aspera *(Willd.) Link	*Nilathulasi* *Thumbai*	Paste of the plant mixed with limestone and applied to catfish bites. The thick roots are used as a tooth brush. Continuous use of a special preparation of this plant for 40 days makes one resistant to some snake poison. Flowers used during religious festivals.
Liliaceae		
*Scilla hyacinthina *(Roth.) Macbr. Contr.	*Narivangayam*	Bulbs used as substitute for onion.
Loranthaceae		
*Dendrophthoe falcata *(L.f.) Etting	*Ottai*	Fruits edible.
Marsileaceae		
*Marsilea quadrifolia *L.	*Aarakeerai*	Leaves used as green vegetable.
Meliaceae		
*Azadirachta indica *A. Juss.	*Veppam*	Tender leaf and bark extract consumed to eliminate stomach worms. Leaf twigs with leaves are tied or hanged in front of the house entrance to keep away evil spirits.
Minispermaceae		
*Tiliacora acuminata *(Lam.) Hook. f. & Thoms	*Perunkkattukodi*	Used as rope or binding bundles fuel woods.
Sapotaceae		
*Mimusops elengi L*.	Magizhamaram	Irulas name for this tree's flower is "Magishampoo". Irulas collect this flower from the forest and store them after they have been dried in the shade.
***Tinospora cordifolia ***(Willd.) Miers	*Seenthil*	Stem and aerial roots ground into a fine powder and consumed with tea or milk. It is used to cure many ailments.
Myrtaceae		
*Syzygium cumini *(L.) Skeels	*Naval*	Bark used for diarrhoea. The extract of bark mixed with goat milk and immediately consumed to stop diarrhoea.
Orchidaceae		
*Cymbidium aloifolium *(L.) Sw.	*Ottai*	Aerial root juice used as ear drop for relieving pain.
Pandanaceae		
*Pandanus fascicularis *Lam.	*Thazahi*	Silt roots used for making fibers and brushes. Fruits occasionally used as vegetable.
Poaceae		
*Bambusa arundinacea *(Retz.) Willd *Cynodon dactylon *(L.) Pers.	*Moongil* *Arugampul*	Young shoots used as food. Stems used to build huts and spiritual ceremonies. Branches dipped in hot oil are used for a head-bath or as a body coolant.
Rhamnaceae		
*Ziziphus mauritiana *Lam.	*Elanthai*	Fruit edible. Powdered bark applied to old wounds.
Rubiaceae		
*Catunaregam spinosa *(Thunb.) Tirveng.	*Kattukoyya*	Fruit is cooked with other vegetables.
Rutaceae		
*Toddalia asiatica *(L.) Lam.	*Surai*	Decoction of leaves and stems used to controls fever. Fresh fruits eaten to reduce fever and headache.
Salvadoraceae		
*Azima tetracantha *Lam. *Salvadora persica *L.	*Sankan* *Vagai*	Leaves ground into pulp with water, the extract is used to treat a fever. Decoction of fruits and roots used to reduce rheumatic pains. Root used as tooth brush. Fruits commonly eaten.
**Sapindaceae**		
*Cardiospermum halicacabum *L. *Sapindus emarginatus *Vahl	*Mudakkathan* *Poovanthi*	Whole plant used for treating rheumatoid arthritis. Wet ground nuts paste used for body wash as a surfactants
Sapotaceae		
*Manilkara hexandra *(Roxb.) Dubard	*Pala maram*	Latex applied on teeth and gums for toothaches. Fruits collected in large quantity and traded in local market for rice, chilli, tobacco, etc.
Scrophulariaceae		
*Peplidium maritimum *(L.f.) Asch.	*Parupu-keerai*	Leaves and tender stems consumed as food.
Simaroubaceae		
*Ailanthus excelsa *Roxb.	*Pekalathi*	A tonic prepared from the bark and leaves for healing after child birth.
Solanaceae		
*Solanum anguivi *Lam.	*Kandan kathiri*	Tender fruits cooked as vegetable and consumed for treating colds, coughs and fever. The tender leaves are made into a paste to treat intestinal worms.
Tamaricaceae		
*Tamarix indica *willd.	*Kattuchaukku*	Extract of leaves used as laxative.
Verbenaceae		
*Gmelina asiatica *L. *Vitex negundo *L.	*Kumalai Nochi*	Fruits used as substitute for soap. Leaves repel mosquitoes. Leaves boiled in water, which is used a bath to relieve body pain.

### Diversity in Irulas TK of medicinal plants

The Irulas revealed ethnotaxa used to treat a variety of illnesses and promote general good health in their communities. TK of plants used to treat 30 illnesses were sorted into 12 usage categories (Table 1), which were re-modified according to Cook [65] and Heinrich [4]. Plant remedies were utilized for various illness such as mosquito repellents, antibiotics, antidotes, appetizers, asthma, blood flow, body pain, body weak or feeble, cold, complementary food, cough, diabetes, diarrhoea, earache, eye pain, fever, general medicine, hydrocoele, hypothermia, intestinal worms, jaundice, leprosy, pregnancy pain, purgative, rheumatism, skin disease, skin-shine, spiritual, toothache and wounds (Table 1). The diversity of ailments in our study may be limited to the common plants in our sample and some of the more common ailments treated in the Irulas villages, including ailments of mainstream people that come to the Irulas to be healed. Native healing practitioners provide a source of income for many Irulas families.

**Figure 4:**
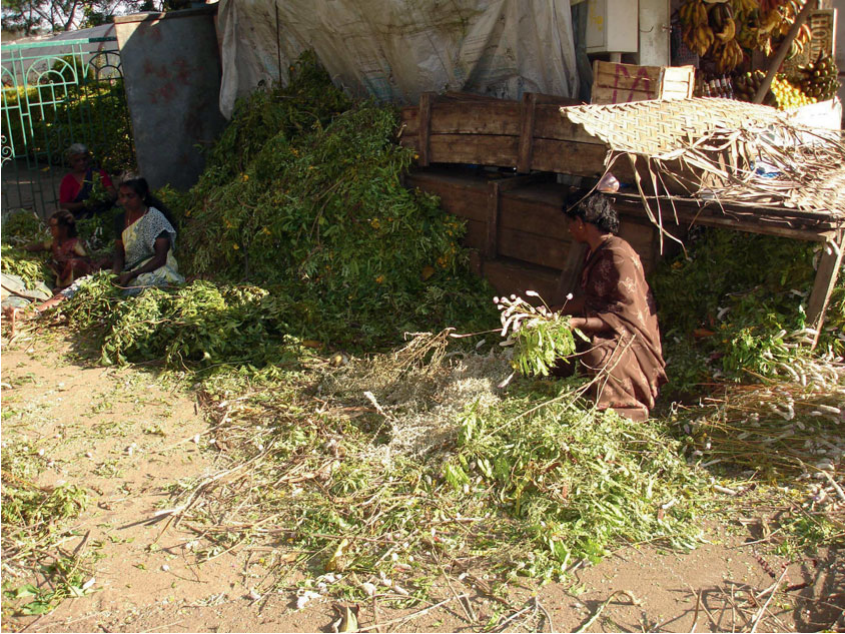
**Collections of ethnotaxa (Kannupila, Pannaipoo, Veppan, Avarai *and Nochi etc*.) for sorting and distribution within the community or trading with other communities**.

**Figure 5:**
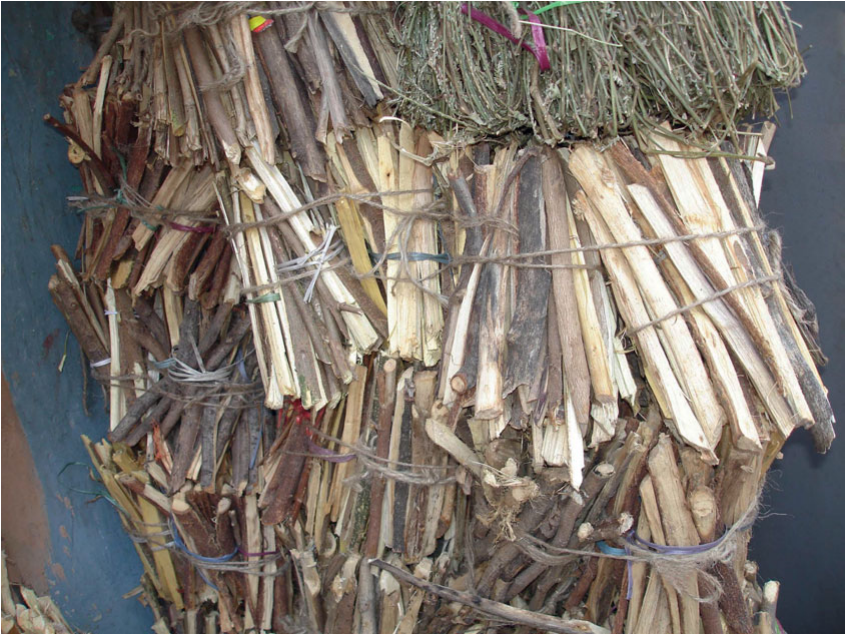
**Bundles of ethnotaxa (*Nagarasi, Veeraii, Thillai, Vellerugu, Thazahi, Vagai etc*.) stored and traded for use as ethnomedicine**.

The Irulas shared the concept 'Neenda aauil', which translates to "living a long healthy life". The Irulas believe that the treatment of ailments can be pre-empted by a healthy life-style. The Irulas routinely consume plants for their vital well being and good health. Previous research indicates other cultures such as the Malasars that put a strong emphasis on daily consumption of plants to promote good health [37], which is supported by other studies that have recommended including the "general health" category in the standardized illness groupings [65]. We included this category because it is an integral part of the Irulas health concept in which healers insist on having plants as part of their diet to maintain good health. The general health category included the largest number of taxa, reports of utility and a relatively high level of consensus (Table 2). We found in our survey that some of the plants used in the general health category are edible to the Irulas (8 species), while others were non-edible (19 species) (Table 3). The great variety of plants that are collected from the wild and distributed among the community (Figure 4) or sent off to local markets for trading with other communities (Figure 5). An ancient tradition of the Irulas is to eat certain plants on a regular basis according to the seasons in order to prevent certain diseases. It is a common practice for the Irulas to consume plants in the wild throughout their daily routine. While hunting, the Irulas grab leaves of *Capparis zeylanica *L. and eat them fresh to stimulate their hunger and increase their desire to hunt. This plant is also served as an appetizer prepared as a dipping paste with pepper, tamarind and garlic. While trekking through the forest the Irulas would pick leaves to eat or chew on young tree branches; the forest is a natural pharmacopeia/grocery store for them.

The young Irulas are also engaged in the use of plants in their daily routine. Not unlike our modern society they encourage good hygiene at a young age. This may include cleaning or brushing your teeth with various types of young branches. However, we found that they combine both cleaning teeth at a young age with an ancient tradition called "priming". The Irulas believe that brushing your teeth with the roots of ***thumbai ***(*Leucas aspera *Willd.) for 40 consecutive days will make you immune to any snake venom. Called "priming", all children were once taught early in life to "prime" with ***thumbai ***before hunting snakes. Presently, very few elders in the Irulas community teach the practice of herbal immune priming to children.

One of the ailments that may be controlled by daily consumption of a particular plant is diabetes. Irulas have an ancient diagnostic system that utilizes their natural surroundings; to determine whether someone has *'Chakari noie' *(diabetes) the person is asked to urinate on the ground to see if ants are attracted to the abnormally high sugar concentration in their urine. The Irulas treat diabetes using an ethnotaxa called *'Sirukurinjan'*, which appears to be *Gymnema sylvestre *in the Asclepiadaceae or "milkweed family". The young, tender, shiny pale green leaves are preferred over the mature leaves, which will also be used. This TK was supported by high consensus among the informants (F_ic _= 1.0). Many Irulas families grow *Gymnema sylvestre *as a climbing vine near their home and it is a household custom to consume one leaf a day. They also regularly add leaves to several of the common curry dishes at least once a week even though the leaves add a bitter taste to the meal. If a young member if the household is diagnosed with *'Chakari noie' *(diabetes), they will be asked to consume 2 to 3 fresh tender leaves every morning before meals with a glass of water for about a year. Other research studies have documented *Gymnema sylvestre *as a natural remedy for diabetes [24,71-78]. We recently recorded that the Malasars use *Gymnema sylvestre *for treating scorpion and rat bites [37], but do not use it to treat diabetes. Perhaps this is due to the fact that *Gymnema sylvestre *is not common at higher altitudes where the Malasars live.

**Figure 6:**
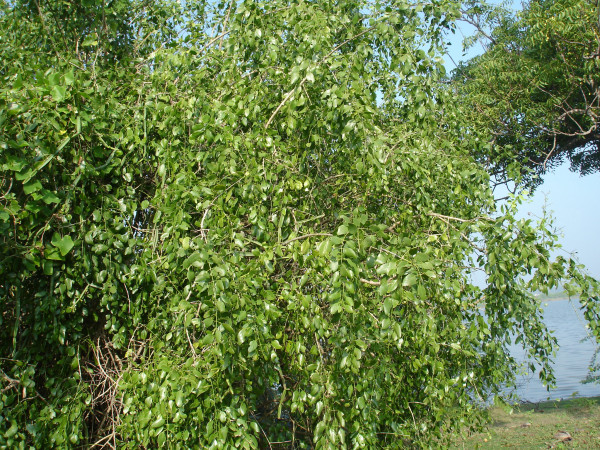
**'*Vagai*' and ancient remedy for '*Moottu vadham*' (rheumatoid arthritis)**.

**Figure 7:**
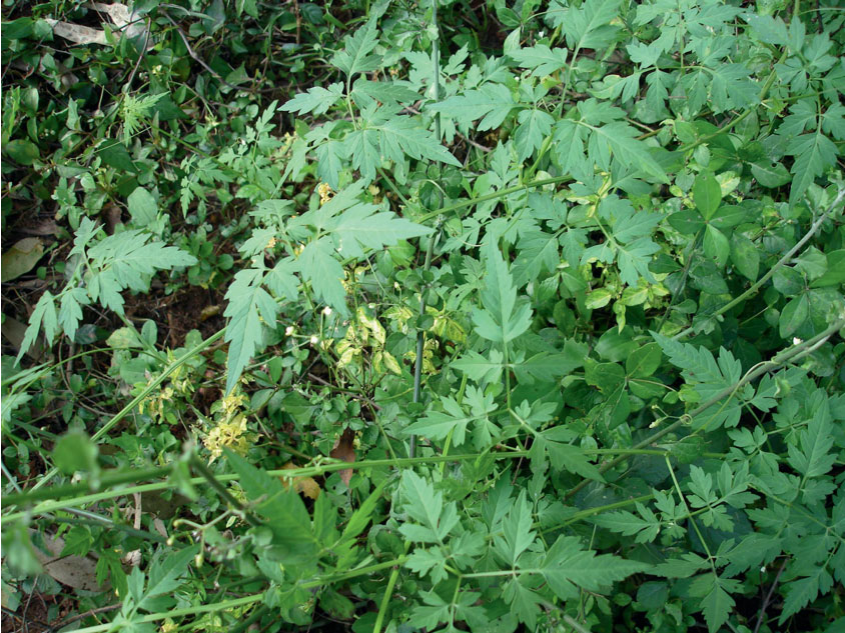
**'*Mudakkathan*' ancient remedy for '*Moottu vadham' *(rheumatoid arthritis)**.

The Irulas treat '*Moottu vadham*' (rheumatoid arthritis) with at least two different ethnotaxa, including '*Vagai*' (Figure 6) and '*Mudakkathan' *(Figure 7) both of which have a high consensus factor for the Irulas (F_ic _= 1.0) within this study and by Malasars (F_ic _= 0.78) [37]. The Malasars treat rheumatic pain using two other plants, *Diplocyclos palmatus *(L.) C. Jeffrey and *Boerhavia diffusa *L. [37]. We identified '*Mudakkathan' *to be *Cardiospermum halicacabum L. sensu lato *('balloon vine') and '*Vagai' *to be *Salvadora persica *L. Previous research by Ragupathy [42] revealed a complex taxonomy of several ethnotaxa for 'balloon vine', which has many associated utilities, including a remedy for joint pain. In many cultures this plant is harvested in backyards for both medicinal and food value. In fact, it provides an income supplement for some families from impoverished communities of third world countries. The paradox is that *Cardiospermum halicacabum *is a poisonous (contains cyanide) invasive plant that agricultural researchers have described as a noxious weed that must be globally eradicated. We are currently investigating genetic variation among the haplotypes of *Cardiospermum halicacabum *and the complex cryptic taxonomy, which consists of several ethnotaxa.

Some modalities are less specific and are treated with a general all purpose plant such as '*Veeraii'*, which means 'vigorous blood'. '*Veeraii' *has reddish sap and is used to treat *'Ratha o'ttam' *(blood circulation problems). We identified this ethnotaxa as *Diospyros ferrea *var. *buxifolia *(Wild.) Bahk. Previous research indicates that Malasars used *'Ratha surai' (Begonia malabarica *Roxb.), which also has reddish sap, to treat blood disorders [37]. We had one record of the Irulas (an isolated mountain community near the Malasars), which used *Begonia malabarica Roxb*. to treat blood cancer. We assume that there are potential possibilities of knowledge sharing and further research is exploring variance in TK with respect different cultures and spatial scales.

**Figure 8:**
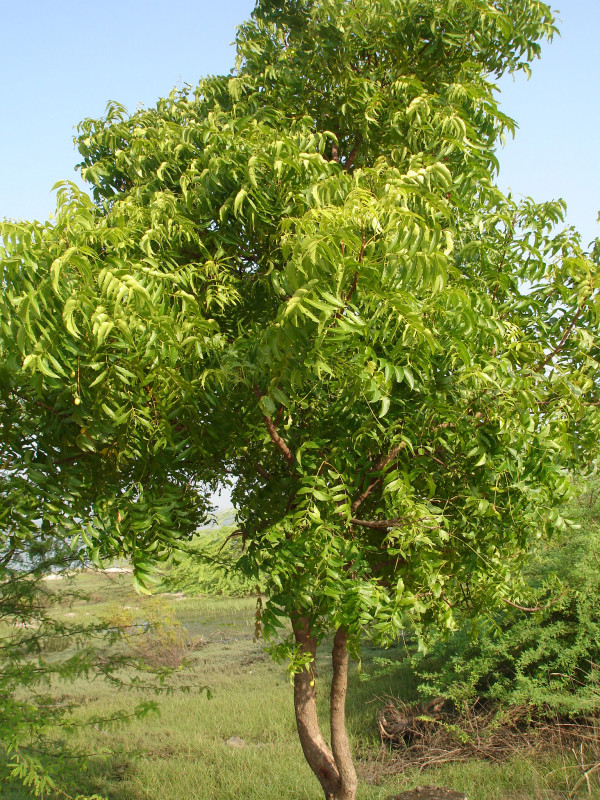
***'Veppam' *is collected as a bunch of twigs tied in a bundle and placed above every household doorway to keep away evil sprits**.

The Irulas are also strong believers in spiritualism for which they utilize many plants. Although we will not report in detail plants used for spiritual reasons it is important to note that there was high consensus (F_ic _= 0.91) among the informants for this utility category. We will briefly describe one application. We noticed that above every household doorway are a bunch of twigs tied in a bundle. This is *'Veppam' *(*Azadirachta indica *A. Juss.; Figure 8) and it is used to keep away evil sprits. Similarly, the Malasars who live in the mountain forest use plants (F_ic _= 0.87), such as *Abrus precatorius *L., *Crotalaria verrucosa *and *Selaginella rupestris*, to repel-evil [37]. There are several ethnotaxa used to repel evil and the preference for a particular ethnotaxa may be related to availability of the plant, but more likely the specific circumstances of the spiritual practice. Most treatments for folk illnesses can be found within cultural references; the Irulas treatment involves praying, communication with the evil sprit and the use of plants.

## Conclusion

Throughout history aboriginal people have been the custodians of biodiversity while sustaining healthy life-styles that utilize natural resources. Unfortunately, their basic requirements sometimes force them into activities such as deforestation for monetary gains, or for extending agricultural activities that lead to a loss of biodiversity, which is the very source of their food and medicines. The Irulas are an excellent example of a culture that has not exploited a highly diverse area (KRF) despite the fact that they harvest their food and medicine from the wild for thousands of years. We have identified the reliable TK concerning the extent of the medicinal utility of plants by the Irulas that can be valorized using a consensus approach. This is the first step toward the recognition of TK in a scientific approach that will enhance society, nutrition, medicine, and resource management, etc. [1]. A quantitative approach provides a means of validation and a measure of confidence. Clear methodology allows the research to be repeated and results to be applied at a larger scale to heal common ailments in society-at-large and use TK to understand biodiversity so that we can develop better management strategies and policies. We are currently conducting a study of the Irulas Traditional Ecological Knowledge (TEK) in order to understand their approach to ecosystem classification, conservation and sustainable utilization of natural resources.

The asymmetry between scientific and TK demands a new approach. We have identified the fact that ethnotaxa are not always synonymous with Linnaean taxonomy [43,79]. In fact, TK has been used to expose cryptic diversity and discover new species that have utility to local cultures and must be considered within broader ecosystem management plans [80,81]. However, these ethno-classification systems are very complicated and require a great deal of time to fully comprehend, reconstruct and utilize. This is a major impediment to engaging ethno-classification and the associated wealth of TK within the design of a comprehensive biodiversity study.

Modern automated identification technology (AIT), such as DNA barcoding, may provide a quick, repeatable and reliable tool for identifying ethnotaxa and variation in cryptic species. DNA barcoding is a method of species identification and recognition using specific regions of DNA sequence data [82,83]. This research can be reviewed on-line via the Canadian Barcode of Life  and the Consortium for the Barcode of Life (CBOL) . Recent development of an AIT system for plants indicates that the efficacy of an AIT system equates with savings in time and resources, while providing quick, reliable identifications [84,85]. We are currently using DNA barcoding to discriminate the cryptic ethno-taxa for nutmegs [86], Acacia [87], Tripogon [81] and within regional floras [85]. We propose that a DNA barcode may be a quick and reliable tool to identify ethnotaxa, which will further legitimize the validity of TK, rendering it testable and ultimately generalisable, mobile and globally meaningful [2].

One of our goals in this research is to document some of the Irulas TK using a science-based consensus approach. We were also able to protect this knowledge within our aboriginal repository of knowledge (ARK) research program and hope to develop educational programs within the Irulas communities. A greater understanding of TK is beginning to emerge based on our research with both the Irulas and Malasars; they believe that a healthy lifestyle is founded on a healthy environment. They chose to share this knowledge with society-at-large in order to promote a global lifestyle of health and environmental sustainability.

## Competing interests

The authors declare that they have no competing interests.

## Authors' contributions

SR and SGN equally contributed to the design, data analysis and writing of this manuscript. Both authors read and approved the final manuscript.
